# 

**Published:** 1908-06-20

**Authors:** 


					?--M*.  '- THE MODERN ^fBXSp*
L
J
hone Number t
ii , . _r 2734 Gerrard.
Oepedele. London. MEDICAL JOURNAL^ ; j
V 3l ^ /
i
Ew SERIES. No. 67, Vol. III. SATURDAY
^*0.1139. Vol. XLIV. OATUKDAY,
June 20th, 1908.
PRICE THREEPENCE.

				

